# Preference of position in the proximity of various sugars revealed by location analysis of *Drosophila melanogaster*

**DOI:** 10.1038/s41598-024-61457-w

**Published:** 2024-05-17

**Authors:** Haruki Kato, Hiroyuki Nakagawa, Chiaki Ishizaki, Jun Tomita, Kazuhiko Kume

**Affiliations:** Department of Neuropharmacology, Graduate School of Pharmaceutical Sciences, Nagoya City University, Tanabe 3-1, Mizuho, Nagoya, Aichi 467-8603 Japan

**Keywords:** Position preference, Nutrition, Starvation, Locomotor activity, Food selectivity, Neuroscience, Circadian rhythms and sleep

## Abstract

Feeding behaviors are determined by two main factors. One is the internal state, such as hunger or previous experiences; the other is external factors, such as sensory stimulation. During starvation, animals must balance food-seeking behavior with energy conservation. The fruit fly, *Drosophila melanogaster*, serves as a useful model for studying food selectivity and various behaviors related to food intake. However, few studies have directly connected food selectivity with other behaviors, such as locomotor activity and sleep. In this study, we report that flies exhibited a preference for specific positions and spent more time in the proximity of sweet sugars, such as sucrose and sucralose, but not non-sweet and nutritious sugars like xylitol and sorbitol. On the other hand, prolonged exposure to sorbitol increased the staying time of flies in the proximity of sorbitol. Additionally, after starvation, flies immediately exhibited a position preference in the proximity of sorbitol. These findings suggest that flies prefer the proximity of sweet food, and starvation alters their preference for nutritious food, which may be beneficial for their survival.

## Introduction

Changing behavior in response to environmental cues and internal physiological conditions is a crucial aspect of survival for living organisms^1^. Particularly in feeding behavior, the assessment of food availability in relation to internal hunger conditions is very important. Several studies using fruit flies have explored this phenomenon through various approaches, including the proboscis extension response (PER)^2^ assay, food intake measurement^3^, and associative memory learning involving odor and food^4^. In addition to food evaluation, investigations into food-seeking behavior and post-food discovery behavior have been undertaken. Previous research has indicated that sweet taste influences searching behavior after food discovery^5^ and dopaminergic signaling in the brain plays a significant role in foraging behavior^6^. Most of these studies have focused on responses to direct food presentation, with only a few examining sleep and locomotor activity in the context of food choices. Furthermore, the monitoring duration in these studies has been limited to short-term intervals ranging from a few minutes up to 24 h^7^. While circadian rhythm and sleep research have provided high-resolution monitoring of fly behavior over longer periods, their primary emphasis has been on changes in circadian rhythm and sleep duration, with limited attention given to food selection in response to nutritional status^7^.

We have previously reported on the relationship between sleep, metabolism, and nutritional status in flies^8–14^. Our research and that of several other laboratories have shown that starvation induces hyperactivity and reduces sleep^15,16^, while sweetness promotes sleep^17^. Sorbitol, a nutritious but non-sweet sugar for flies, triggers behavior similar to that observed during starvation. However, flies are known to form associative memories between odors and nutritional content^18,19^, and they selectively feed on nutrients in the 2-choice and the CAFÉ assay^20–22^. Furthermore, survival has been demonstrated in flies fed exclusively on sorbitol^15^.

In this study, we used a multibeam monitor to assess long-term behavior under various food conditions, employing a straightforward and high-resolution method to record fly behavior. By analyzing the precise time series data of position and activity of flies within a tube, we identified their preference for locations in proximity to sweet food sources. Moreover, this preference changed under starvation conditions.

## Results

### Flies stayed for a longer time in the proximity of sweet sugar food, but not that of non-sweet and nutritious sugar food

**Figure 1:**
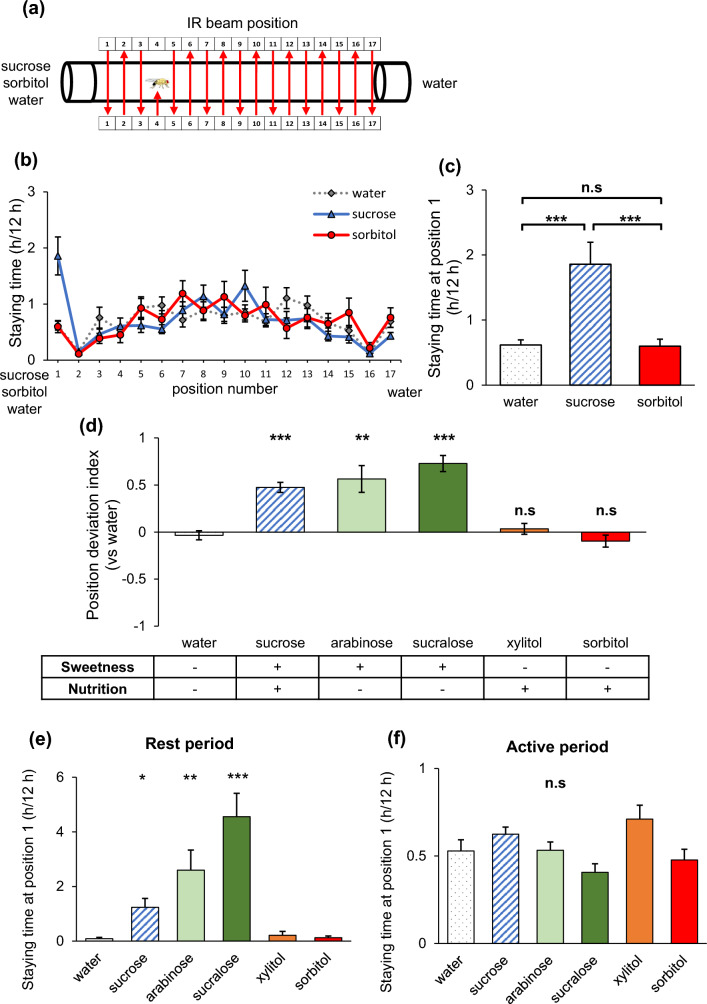
Sweet sugars induced flies to stay in the proximity of food during subjective daytime, while non-sweet sugars did not. (**a**) Schematic diagram of the multibeam monitor experiment. The preference for sugar was assessed based on the position of the flies. Each sugar was prepared at 5% concentration and dissolved in 1% agar. Sugar food was placed on one end (position 1 side), and water food was placed on the other end (position 17 side). (**b**) Staying time in individual channels and (**c**) at position 1 which was closest to the water food, sucrose food, or sorbitol food. Each data is presented as mean ± SEM (n = 29, 32, 16). ***p < 0.001, n.s: not significant; Steel–Dwass test. (**d**) PDI, which represents the deviation of staying time in response to sugar compared to water food during subjective daytime. (**e**, **f**) Staying time at position 1 during (**e**) the rest period and (**f**) the active period. Each data is presented as mean ± SEM (n = 29, 32, 11, 14, 16, 16). *p < 0.05, **p < 0.01, ***p < 0.001, n.s: not significant vs agar; Steel test.

To measure the locomotor activity and location preference of flies in a tube where they could choose between two types of food on both sides, we introduced a fly into the tube through the hole at the center and recorded its position while it had access to 5% sugar food on one end (position 1 side) and water food on the other end (position 17 side), (Fig. 1a). During the subjective daytime, the staying time at position 1, which was close to sucrose food, was significantly longer than that at positions close to water food and sorbitol food (Fig. 1b, c). To evaluate the position preference, we calculated the Position Deviation Index (PDI) for each type of food, which is the ratio of the staying time between position 1 (near sucrose and other sugar foods) and position 17 (near water food) within one tube divided by the sum of the two. The PDI for sweet sugar food, such as sucrose, arabinose, and sucralose, was significantly higher than that for water food on both sides, regardless of the nutritional value of the sugar (Fig. 1d). However, the PDI for non-sweet and nutritious sugar food, such as xylitol and sorbitol, did not significantly differ from that of water food on both sides (Fig. 1d). Further analysis involved classifying staying times into two groups: rest periods and active periods. When a fly stayed in the same position for equal to or longer than 5 min, a common definition of sleep in flies, it was classified as a rest period. Conversely, when a fly stayed in the same position for less than 5 min, it was classified as an active period. In rest periods, the staying time near sucrose, arabinose, and sucralose food was longer compared to water food (Fig. 1e), while the staying time near xylitol and sorbitol food did not differ from that near water food. Conversely, during active periods, there were no significant differences in the staying time near water food and other sugars (Fig. 1f). A similar analysis was conducted during subjective nighttime, and it revealed a similar pattern (Sup. Fig. 1). Consequently, all subsequent analyses were exclusively based on data collected during subjective daytime. These results suggest that sweetness information increases the staying time near food, especially during rest periods of the subjective daytime, which can be considered as daytime sleep, while nutritional information itself does not significantly influence the staying time.

### Staying time in the proximity of sorbitol increased over time

**Figure 2:**
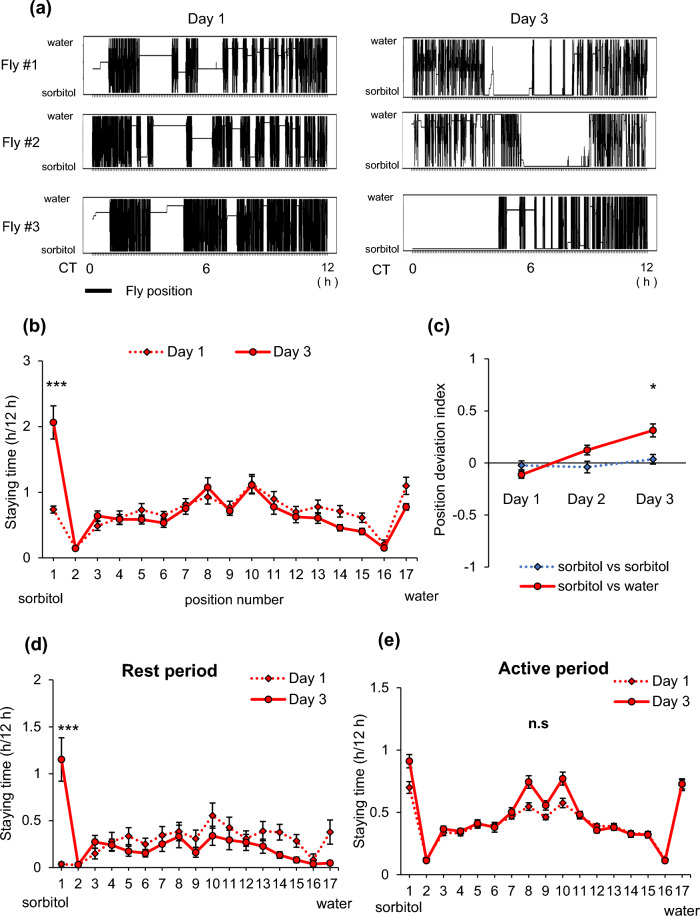
Staying time in the proximity of sorbitol during the subjective daytime increased with time. (**a–e**) Behavioral changes in flies with time when fed sorbitol food during daytime. (**a**) Examples of tracing the staying position of flies during the subjective daytime of day 1 (left) and day 3 (right) of the measurement. The vertical axis indicates the location of the flies, with the lower side indicating proximity to sorbitol food and the upper side indicating proximity to water food. (**b**) The change in staying time in the tube during the subjective daytime of day 1 and day 3. (**c**) The change in PDI. (**d**) Staying time at position 1 during the rest period and (**e**) the active period. Sorbitol was prepared at 5% concentration and dissolved in 1% agar. Each data is presented as mean ± SEM (sorbitol vs sorbitol: n = 51, sorbitol vs agar: n = 42). *p < 0.05, ***p < 0.001, n.s: not significant; vs. day1; Steel–Dwass test (**b**, **d**, **e**), vs. “sorbitol vs sorbitol”; Steel–Dwass test (**c**).

Next, we conducted long-term measurements to observe temporal changes in fly behavior, especially in response to sorbitol food. Figure 2a shows examples of the actual tracing of fly locations in tubes containing sorbitol and water food during the subjective daytime on day 1 and day 3. The vertical lines indicate fly movement, while the horizontal lines represent prolonged staying at one position. On day 1, there were no apparent preferred fixed positions where flies stayed for a long time, but on day 3, flies tended to stay for extended periods in the proximity of sorbitol food (indicated by the downside of the plot). The staying time in the proximity of sorbitol food during the subjective daytime on day 3 was significantly longer compared to day 1 (Fig. 2b), and the Position Deviation Index (PDI) for sorbitol food also increased (Fig. 2c). Comparison of position 1 and position 17 showed no significant difference on day 1 (Fig. 2b, position 1 vs position 17 on Day 1, *p* = 0.97), but a significant difference on day 3 (Fig. 2b, position 1 vs position 17 on Day 3, *p* < 0.001). Additionally, the staying time in the proximity of sorbitol food during rest periods showed an increase similar to that of sweet food in Fig. 1e (position 1 in Fig. 2d). While the staying time during active periods also exhibited a slight increase, it did not reach statistical significance (Fig. 2e). We excluded flies that died before or on day 3 from the analysis for both day 1 and day 3 data. We analyzed the data from day 1 for the flies that were excluded from the analysis. However, there were no significant differences in the staying time in the proximity of sorbitol food or the PDI for sorbitol food on day 1 between flies that survived and those that died (Sup. Fig. 2). We also conducted long-term measurements using sucrose food and found that the staying time in the proximity of sucrose was significantly longer from day 1 and tended to increase further on day 3 (Sup. Fig. 4). We principally used male flies in this study since most of our previous works used males, but we confirmed female flies also showed similar characteristics. They showed preference for sorbitol on day 3, but not on day 1 and PDI for sorbitol increased from day 1 to 3 (Sup. Fig. 5). In addition, we performed a similar experiment under conditions with 1% sucralose added on both sides to eliminate the effect of sweetness. On day 1, in comparison between position 1 and position 17, the staying time at position 1 was significantly shorter than position 17 (Sup. Fig. 6a, position 1 vs position 17 on Day 1, *p* < 0.001) meaning flies avoided sorbitol with sucralose food compared to the sucralose-only food. On the other hand, there was no significant difference on day 3 (Sup. Fig. 6a, position 1 vs position 17 on Day 3, *p* = 1.00), indicating the preference for sorbitol increased. Furthermore, the staying time at position 1 significantly increased on day 3 compared to day 1 (Sup. Fig. 6a). These trends were more robust in the rest period (Sup. Fig. 6c, d). These data suggest that feeding sorbitol has no effect on the staying time in the proximity at first, but over time it induces a staying time in its proximity.

### Starvation increased the staying time in the proximity of sorbitol

**Figure 3:**
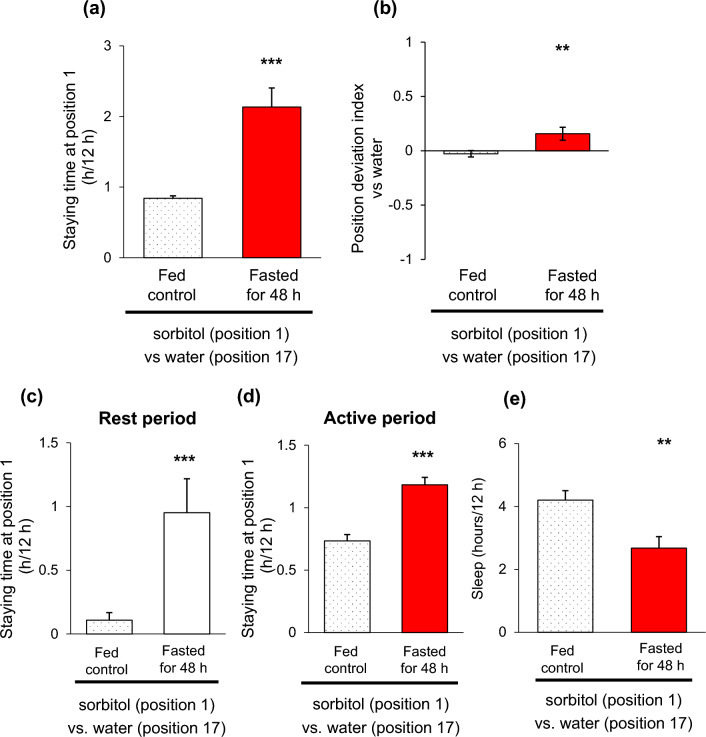
Starvation increased the staying time in the proximity of sorbitol. (**a–e**) Behavioral changes were measured after 48 h of starvation when flies were fed with sorbitol food during the subjective daytime of day 1. As a control group, we used flies fed with 5% sucrose + 1% agar for 48 h. Staying time at position 1 (**a**), PDI to sorbitol food (**b**), staying time at position 1 during the rest period (**c**) and the active period (**d**), and amount of sleep (**e**). Sorbitol was prepared at 5% and dissolved in 1% agar. Each data is presented as mean ± SEM (n = 48, 36). **p < 0.01, ***p < 0.001, Mann–Whitney U test (**d**, **e**).

In previous studies, it has been reported that the staying time in the proximity of sucrose food increases after starvation^7^ and that temporary sleep duration increases after feeding^23,24^. When flies were presented with a choice of sorbitol or water food in the tube, they displayed reduced sleep, resembling the effects of starvation (Sup. Fig. 3). Therefore, we hypothesized that the change in staying position over time was due to a starvation-like state, even in the presence of sorbitol food. Consequently, we conducted measurements after subjecting the flies to a 48-h starvation period. The starved flies exhibited increased staying time in the proximity of sorbitol food during the subjective daytime of day 1, compared to the control group fed with 5% sucrose (Fig. 3a). Additionally, the PDI for sorbitol food significantly increased (Fig. 3b). The increase in staying time in the proximity of sorbitol food was observed both during rest periods and active periods (Fig. 3c, d). Sleep duration in the post-starved flies remained significantly shorter than in the fed control flies (Fig. 3e). We also examined the effects of starvation on sucrose preference and found that starvation increased the staying time in the proximity and PDI for sucrose food, which was particularly pronounced during the rest period (Sup. Fig. 7a–d). In addition, the amount of sleep recovered to the same level as that of the control group (Sup. Fig. 7e). These results suggest that starvation leads flies to spend more time in the proximity of sorbitol.

### With prolonged starvation time, flies acquired a preference for sorbitol

**Figure 4:**
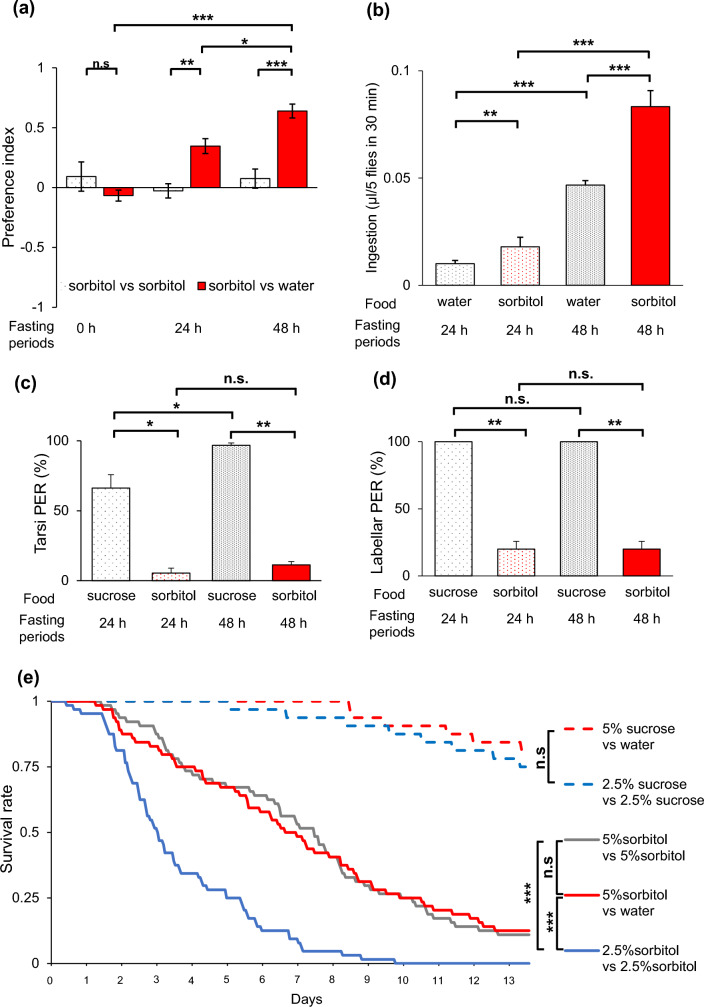
Preference for sorbitol is acquired with prolonged starvation time. (**a**–**d**) Several assays measured behavior toward sorbitol during prolonged starvation. (**a**) 2-choice assay for sorbitol food after 0, 24, and 48 h of starvation (n = 10, 12, 10, 12, 10, 12). (**b**) Water food or sorbitol food consumption after 24 and 48 h of starvation (n = 73, 72, 73, 71). (**c**, **d**) Tarsi PER (c, n = 3) and Labellar PER (d, n = 3) for sucrose or sorbitol when flies were starved for 24 and 48 h. Each data is presented as mean ± SEM. *p < 0.05, **p < 0.01, ***p < 0.001, n.s: not significant; one-way ANOVA followed by Tukey–Kramer post hoc test (**a**), Steel–Dwass test (**b**), two-way ANOVA followed by simple effect test (**c**, **d**). (**e**) Survival was measured for the group given a choice of sorbitol or not (n = 32, 32, 64, 64, 64). Survival rates are plotted every hour. Sucrose or sorbitol was prepared at each concentration and dissolved in 1% agar. ***p < 0.01, n.s: not significant; log rank test with Bonferroni correction.

To investigate the relationship between the perception of sorbitol and this behavioral change towards sorbitol food, we examined the selectivity and sensitivity to sorbitol in flies depending on the starvation period. We prepared two starvation periods: 24 h, during which most flies survived, and 48 h, during which about 60% of the flies survived^17^. First, we conducted a 2-choice assay to measure the selectivity for food, by quantifying the food ingested by flies based on its color. The selectivity for sorbitol, which was not evident without starvation, emerged and significantly increased with the duration of starvation (Fig. 4a). Moreover, the consumption of sorbitol also increased as the duration of starvation extended, as observed in the feeding assay. After 48 h of starvation, the consumption of sorbitol increased significantly compared to water food. After 24 h of starvation, there was a slight increase in sorbitol ingestion (Fig. 4b). We conducted a similar experiment with sucrose and found that the amount of food consumption increased with prolonged fasting periods, and the amount of sucrose consumption was 5 to 10 times higher than that of sorbitol (Sup. Fig. 8).

Secondly, we performed a proboscis extension (PER) assay to measure the sensitivity to sorbitol, which evaluates the immediate response of flies presented with sugars. The sensitivity to sucrose with 48 h of starvation increased compared with 24 h of starvation period. On the other hand, the sensitivity of both legs and proboscis to sorbitol solution remained low even after 48 h of starvation (Fig. 4c, d). These results suggest that flies acquire a preference for sorbitol as the starvation period prolongs, and this change is not attributed to alterations in peripheral taste reception. Additionally, we observed no difference in survival between the group with 5% sorbitol food on one side of the tube and water food on the other side, compared to the group with 5% sorbitol food on both sides. However, there was a significant difference in survival rates between the group with 2.5% sorbitol food on both ends (Fig. 4e).

## Discussion

This study introduced a novel and simple method to examine the behavioral preference of fly locations and demonstrated that flies alter their prolonged stay positions during the daytime, depending on the nature of the food and their internal hunger state. We found that sweetness, but not nutrition, led flies to stay longer in the proximity of food. However, with starvation, nutrition also played a role in prolonging their stay. Interestingly, the changes in preference for the position after starvation appeared to involve mechanisms distinct from peripheral sensory input.

Specifically, in this study, we used the staying time at the position in the proximity of the food, along with PDI (Position Deviation Index), a novel index as an indicator of food preference. Previous research on sugar preference mainly focused on examining feeding amounts of sugars or assessing selectivity for sugar based on food consumption. Even experiments with video analysis were limited to short durations^5,25^ with some extending up to 24 h^7^. In contrast, our experimental approach allowed for the observation of long-term changes over several days by providing two types of food at each end of the tube and continuously tracking the location of the fly using a multibeam monitor. This enabled us to easily analyze the changes in sleep and activity with long-term and high temporal resolution. Previous studies have reported that flies tend to rest near the food in 96% of all rest bouts^26^. However, our study revealed that although flies did tend to rest longer near food sources compared to agar, this is not much different at night, when they generally sleep longer than during the day (Sup. Fig. 1). We speculate that this variation could be attributed to differences in the food type; for instance, Hendricks et al.^26^, employed standard food in their research, and olfactory cues might also influence the choice of resting location. Furthermore, materials such as cotton^27^, yarn^26^, or parafilm^28^ were used on the opposing side in other studies. In our experiments, we placed agar on both sides, which may have contributed to the differences observed compared to previous studies. Additionally, we observed that flies tended to spend more time at the center of the tube, and we speculated that this behavior was influenced by the presence of the small air hole located at the center of the tube.

During the subjective daytime under constant dark condition, we observed that flies spent a longer time in the proximity of sweet sugar foods, particularly during stays lasting longer than 5 min, which we defined as rest periods (Figs. 1e, f, 2d, e). We are interested in investigating fly behaviors under light–dark conditions; however, currently, we face technical limitations preventing us from conducting similar assays in the presence of light. Light exposure induces deviations in fly positioning, and we have not yet been able to eliminate this effect of light in our experiments.

Previous studies such as Mahishi et al.^7^, reported that starvation was required for staying in the proximity of sucrose. However, in our results, the staying time in the proximity of sucrose was longer than that of water food from the first day of the measurement. The reason for this difference is not entirely clear, but we speculate that there may be variations between different fly stocks. For instance, the *w*^*1118*^ strain in our lab has been reported to spend most of its time closer to the sucrose end of the tube^27^. On the other hand, our examination of Canton S flies from our lab stocks showed a weaker preference for a specific position without starvation (data not shown). Additionally, the results in Fig. 1 indicated a trend of longer staying time at position 1 for arabinose and sucralose compared to sucrose, which suggests that starvation may contribute to the longer staying time in the proximity of food, especially since arabinose and sucralose are not nutritious to flies. Furthermore, our results (Figs. 3, 4) indicated that preference towards sorbitol was also influenced by starvation. These findings indicate that while flies do exhibit a preference for proximity to sweet substances under normal conditions, starvation further enhances this tendency, leading them to stay in the proximity of nutrition along with their preference for sweetness. During starvation, flies may have an adaptive strategy to conserve energy while still maintaining access to food by staying in the proximity of it. Flies can utilize sorbitol as a nutrient but do not perceive it as sweet, and our previous study and this study showed that flies reduce sleep when fed with sorbitol (Hasegawa et al.^17^) (Sup. Fig. 3). Since sucrose recovered sleep amount after starvation, it is suggested that sorbitol alone was not sufficient as a nutrient (Sup. Fig. 7e). Although sleep is often reduced during starvation, previous research has shown that tolerance to starvation is positively correlated with the amount and length of sleep bouts^29^. In addition, flies increase their activity time but decrease the distance they travel when they are starved^30^. During starvation, flies may conserve energy by staying in that location. Therefore, during starvation, flies appear to adopt a dual strategy: actively searching for food while also conserving energy by staying in the proximity of the found food. In nature, flies may evaluate food based on its sweetness and nutrients and decide whether to stay there or to look for other food depending on their internal hunger status.

The PDI for sorbitol food in flies after 48-h starvation (Fig. 3b) was smaller than that in flies fed with sorbitol food for two days (Fig. 2b). While the degree of starvation itself is expected to be stronger in the flies starved for two days, there may be an additional mechanism contributing to the enhancement of preference. It is possible that flies form associative memories with information about the location of food and the type of food they eat^31^, or they may acquire synergistic preferences through repeated experiences of finding food during starvation.

Flies have been reported to exhibit reduced sensitivity to bitter tastes and increased sensitivity to sweet tastes when they are starved^1,32^. Previous studies have shown that flies do not exhibit proboscis extension response (PER), an immediate response to sorbitol from peripheral taste receptor input^18,19,21^. Sweet taste information has been associated with short-term memory, while nutritional information has been linked to long-term memory, and these memory transmissions are mediated by specific clusters of dopamine neurons (PAM neurons)^33^. Based on these findings, we hypothesized that flies detect the nutritional information of sorbitol during starvation through a post-ingestive effect, leading to the acquisition of a preference for sorbitol. Moreover, the weak response in the PER assay with prolonged starvation time suggests that peripheral taste receptors do not play a major role in sensitivity to sorbitol after starvation (Fig. 4c, d). On the other hand, taste receptors are also expressed in the brain^34–36^ and gut^37^. These receptors may be involved in the behavior toward sorbitol.

In Fig. 4e, we conducted an experiment based on the findings from the multibeam monitor analysis, in which flies were presented with a choice of food at both ends of the tube. Assuming that the flies provided with the choice of 5% sorbitol food and agar would consume the food at both ends equally, the survival curve was expected to resemble that of the group exposed to 2.5% sorbitol food on both sides. However, in actuality, the survival curve exhibited a closer resemblance to that of the group supplied with 5% sorbitol food on both sides. This result suggests that the flies selectively ingest the 5% sorbitol food and consequently achieve prolonged survival.

The metabolic pathway involving sorbitol is known as the polyol pathway, in which glucose is converted to fructose via sorbitol, serving as a glucose-sensing system^38^. When sorbitol is ingested, it is converted into fructose through this pathway, phosphorylated by hexokinase, and then enters the glycolytic pathway to yield energy^39^. Flies possess *Gr43a* as a fructose receptor^39^. Gr43a receptor was reported to be expressed in the brain as well as in the peripheral tissues and to function as sensors of nutrition^39,40^. There are also reports suggesting that neurons expressing Gr43a receptor regulate sleep^17^, and fructose-Gr43a pathway may be involved in the preference of position in the proximity of sorbitol.

In conclusion, our study revealed that flies exhibit a preference for their staying position in the proximity of food, which is influenced by their internal satiety state and can be effectively examined using a position recording method with a multibeam monitor.

## Materials and methods

### Fly strains and maintenance

Flies (*Drosophila melanogaster*) were reared on standard food consisting of cornmeal, yeast, glucose, wheat germ, and agar at a temperature of 25 °C under a 12-h light:12-h dark (LD) cycle, as previously described^41^. The *w*^*1118*^ (2202u) stock was originated from the M. Saitoe laboratory^42^ (Tokyo Metropolitan Institute of Medical Science, Tokyo, Japan), and all stocks have been maintained in our laboratory for several years.

### Multibeam monitor analysis

The preference of individual flies for food and their spatial location was assessed using an infrared (IR) multibeam monitor MB5 (Trikinetics, USA). The MB5 consists of 17 infrared beams across the tube, allowing us to capture detailed fly behavior and record their positions as numbers representing the 17 positions between the two ends of the tube (Fig. 1a). Unless otherwise specified, 2–7 days old male flies were used for the experiments. Female flies, 2–7 days old were used regardless of their virginity. Flies were introduced into plastic tubes (inside diameter, 3 mm; length, 65 mm) containing either agar medium (1% agar) or sugar-agar medium (5% sugar and 1% agar) on each side of the tube. Flies were gently placed through a small hole in the center of the tube, and the hole was covered with parafilm. To ensure proper ventilation, a small air hole was drilled using a thin pin vice. These preparatory procedures were conducted at Zeitgeber Time (ZT) 7–12 on the day before the commencement of measurements, and the actual data collection started at ZT0 on the following day. The data collection was carried out for 2 days (Fig. 1) or 4 days (Fig. 2) under constant dark (DD) conditions to minimize the influence of visual inputs. Flies that did not cross the center IR beam after 1 day (Fig. 1) or 3.5 days (Fig. 2) from the beginning of the measurement were excluded from the analysis, considering them as flies that had died during the experiment. All data were recorded at 2-s intervals, utilizing the "Position" and "Count" functions. The data were organized into 12-h intervals to analyze fly behavior during each period. Unless otherwise specified, data from the subjective day (CT0-12) were used for the analysis.

Using the “Position” function, we calculated the staying time at each position in the tube. The multibeam monitor, equipped with 17 infrared detectors, allows for the measurement of the fly's location by dividing the space within the tube into sections from 1 to 17. Using the staying time at positions 1 and 17, which are closest to the two ends of the food, the Position Deviation Index (PDI) was calculated. PDI was calculated as follows [(Staying time at position 1)−(Staying time at position 17)/(Staying time in position 1) + (Staying time at position 17)]. Flies that did not stay at position 1 and position 17 for 12 h were excluded from the PDI calculation. When sugar food was placed on one side only, the food was placed on the side of position 1.

The analysis was divided into two distinct periods: the rest period, defined as the interval during which the position remained unchanged for 5 min or longer, and the active period, defined as the interval during which the position changed within 5 min. The “Count” function was used to measure sleep. Sleep was defined as an interval during which the count was not observed for longer than 5 min. Exceptionally, in Sup. Fig. 5c, all the flies did not stay at position 2 in rest period so that we excluded the data from the statistical analysis.

In the experiment involving starvation periods (Fig. 3), flies were introduced into the tubes between ZT 22 and ZT 24, and the actual experiment commenced at ZT 0. Before the experiment, the flies in the starvation group were kept in vials containing 1% agar, while the control group flies were provided with unrestricted access to a diet consisting of 5% sucrose + 1% agar food. The data analysis was conducted using the R programming language with custom codes.

### Feeding assay

The feeding assay was adapted from previous studies^18,43^. 2–8 days old male flies were used for the experiments. A group of 30–40 flies was subjected to a starvation period of either 24 h or 48 h in a vial containing 1% agar. Subsequently, the starved flies were transferred into vials containing 5% sugar + 1% agar food supplemented with 1% blue dye No. 1 (Osaka food color, Japan) and allowed to feed freely for 30 min. Flies that had died before the start of the assay were excluded. After the feeding period, flies were then collected by 5 flies under cold anesthesia. The collected flies were homogenized in 500 µl of PBS containing 0.01% Triton X-100 and centrifuged twice at 13,000 rpm (10,000 g) for 25 min. The resulting supernatant was analyzed for absorbance at 630 nm using a plate reader (Nivo 3S, Perkin Elmer, USA). The absorbance obtained from flies fed with 1% agar without blue dye No. 1 was subtracted from the measured absorbance, and the food consumption per group of 5 flies was calculated based on the standard curve (R^2^ > 0.99).

### Two-choice assay

The two-choice assay was modified from previous studies^22,43^. 2–8 days old male flies were used. A group of approximately 40 flies underwent a starvation period of either 24 h or 48 h in a vial containing 2 ml of 1% agar at the bottom. Flies that were not subjected to starvation were maintained on standard food. About 40 flies were then anesthetized with cold and placed in a microplate (60-well HLA Terasaki plate, Greiner). Each well in the microplate was filled alternately with either 5% sugar + 1% agar food or 1% agar. To distinguish the two food types, the 5% sugar + 1% agar food was colored with 1.25 mg/ml blue dye No. 1 (Osaka food color, Japan), while the 1% agar food was colored with 2.5 mg/ml red dye No. 2 (TCI, Japan). The microplates were wrapped in aluminum foil to create dark conditions, and the flies were allowed to feed freely for 2 h. Subsequently, the flies were frozen. The preference for sugar was later determined and calculated based on the color of the abdomen. The color of the abdomen was categorized into blue, red, purple, and colorless (indicating no color consumption). The preference index (PI) was calculated using the following formula: [(number of flies with colored abdomens that consumed only sugar food)−(number of flies with colored abdomens that consumed only agar food)]/(total number of blue, red, and purple flies). As a control group, we employed 5% sugar + 1% agar food with each color. To eliminate any potential preference for a specific color, half of the experiments were conducted with the colors reversed, maintaining the food types, but changing the color assigned to each food type.

### PER assay

The PER assay was modified from previous study^1,2,18,43^. 2–8 days old male flies were used. A group of approximately 30–40 flies was subjected to a starvation period of either 24 h or 48 h in a vial containing 2 ml of 1% agar at the bottom. In the Tarsi PER assay, sugar solution was presented to the flies on their front legs, while in the Labellar PER assay, sugar solution was presented to the flies on their proboscis. For the Tarsi PER assay, cold-anesthetized flies were affixed to glass slides using water glue and left for 1 h to recover from anesthesia. Each experiment consisted of 20–30 flies per group, and flies that died or escaped during the experiment were excluded from the overall analysis. To present the sugar solution, a P20 micropipette was used to extrude droplets. Initially, water was presented, and flies were allowed to drink water until no further proboscis extension was observed. Flies that continued to respond to water for longer than 5 min were excluded. Next, a 3 M (1 g/1 ml) sucrose solution was provided as a positive control, and flies that did not extend their proboscis in response to sucrose were also excluded. Subsequently, the flies were alternately presented with water, 5% sorbitol solution, water, and 5% sucrose solution, and their proboscis extension response was recorded. For the Labellar PER assay, flies were fixed into the P20 micropipette tip so that only their heads were exposed. About 10 flies per group were prepared per experiment. A paper cloth (KimWipe, Japan) with a pointed tip was used to present the solution to the flies. After feeding enough water, flies were presented in the same order as in the Tarsi PER assay. Only full extensions of proboscis were counted. Sugar was presented for two cycles, and flies that responded at least once were recorded. Multiple experiments were conducted, and the percentage of flies responding in each experiment was calculated. n = 3 means the data from 3 independent experiments.

### Survival analysis

3–8 days old male flies were used. The Drosophila Activity Monitor (DAM) from Trikinetics (USA) was employed to measure the survival of individual flies when given a choice of food. Plastic tubes with an air hole drilled at one end (inside diameter, 3 mm; length, 65 mm) were filled with food (sugar + 1% agar or 1% agar) to the center (half of the tubes). A fly was introduced into one of the tubes, and the second tube containing either the same or different agar food was connected using cellophane tape. This configuration created a similar length of space as having agar food on both sides of a single tube and was set up in the DAM. As with the multibeam experiment, these preparations were performed at ZT 7-12, on the day before the start of the measurement, and the actual measurement commenced at ZT 0 on the following day. To eliminate any potential visual effects, the experiment was conducted under constant darkness (DD) conditions. The measurements were carried out for 14 days. Fly behavior was observed hourly after the start of the measurement, and the death of a fly was recorded as the time when the last activity counts were observed. As with the multibeam experiment, flies that exhibited the last activity count at 13.5 days or later (12 h before the end of the measurement) were considered to have survived until the end of the experiment and were censored when the last counts were recorded. The survival analysis allowed us to assess the longevity and overall survival rates of the flies in response to the different food choices provided in the DAM setup under constant darkness conditions.

### Statistical analysis

Data were analyzed as described in the figure legends using Microsoft Excel and R 3.6.3 (https://www.R-project.org/). In all bar graphs, data are represented as mean ± SEM. Based on the results of the Shapiro–Wilk test, a one-way or two-way analysis of variance (ANOVA) was performed if the distribution was normal, and a post hoc test was conducted for each comparison. If the distribution was not normal, the Mann–Whitney U test was used for two-group comparisons, and the Steel–Dwass test or Steel's test was used for multi-group comparisons. The range of sample sizes was set based on previous studies with similar experiments, and multiple experiments were conducted to meet that sample size.

### Supplementary Information

Supplementary Information 1.

Supplementary Information 2.

Supplementary Legends.

Supplementary Figures.

## Data Availability

The processed datasets used and/or analysed during the current study are included in this published article and its supplementary information files, and the raw original datasets are available from the corresponding author on reasonable request.
