# Supplementary material for: Preference of position in the proximity of various sugars revealed by location analysis of Drosophila melanogaster
Source: Sci Rep. 2024 May 17;14:11285. doi: 10.1038/s41598-024-61457-w (PMC11101431; doi:10.1038/s41598-024-61457-w)
Supplement: Supplementary file 3 — Supplementary Legends. [file 41598_2024_61457_MOESM3_ESM.docx]

**Supplementary Figure Legends**

**Sup. Fig. 1 The similar trend was observed at subjective nighttime as at subjective daytime.**

(a) Staying time in individual channels and (b) at position 1in water food, sucrose food, or sorbitol food. The used flies were the same as Fig. 1. Each data is presented as mean ± SEM (n =29, 32, 16). **p*＜0.05, n.s: not significant; Steel-Dwass test. (c) PDI during subjective nighttime. Each data is presented as mean ± SEM (n = 29,31,11,13,16,16). ****p*＜0.001, n.s: not significant vs agar; Steel test. (d, e) Staying time at position 1 during (d) the rest period and (e) the active period. Each data is presented as mean ± SEM (n = 29, 32, 11, 14, 16, 16). ***p*＜0.01, ****p*＜0.001, n.s: not significant vs agar; Steel test.

**Sup. Fig. 2 Prolonged staying time in the proximity of sorbitol was not innate.**

(a) Staying time at position 1 and (b) PDI to sorbitol food in subjective daytime of day 1 for flies that died and those that survived during the measurement in Fig. 2. Sorbitol was prepared at 5% concentration and dissolved in 1% agar. Each data is presented as mean ± SEM (n = 22, 42). n.s: not significant; Mann-Whitney U test.

**Sup. Fig. 3 Despite the ingestion of sorbitol, flies remained hyperactive and decreased the amount of sleep.**

The amount of sleep every 24 hours in Fig. 2. Sorbitol was prepared at 5% and dissolved in 1% agar. Each data is presented as mean ± SEM (n = 42). n.s: not significant **p*＜0.05, ****p*＜0.001; Steel-Dwass test.

**Sup. Fig. 4 Staying time in the proximity of sucrose during the subjective daytime.**

(a-d) Behavioral changes in flies with time when fed sucrose food during daytime. (a) The change in staying time in the tube during the subjective daytime of day 1 and day 3. (b) The change in PDI. (c) Staying time at position 1 during the rest period and (d) the active period. sucrose was prepared at 5% concentration and dissolved in 1% agar. Each data is presented as mean ± SEM (n = 32). **p*＜0.05, ****p*＜0.001, n.s: not significant; vs. day1; Steel-Dwass test (a, c, e), vs. “sucrose vs sucrose”; Steel-Dwass test (b).

**Sup. Fig. 5 Staying time in the proximity of sorbitol during the subjective daytime also increased with time in female flies.**

(a-d) Behavioral changes in female flies with time when fed sorbitol food during daytime. (a) The change in staying time in the tube during the subjective daytime of day 1 and day 3. (b) The change in PDI. (c) Staying time at position 1 during the rest period and (d) the active period. Sorbitol was prepared at 5% concentration and dissolved in 1% agar. Each data is presented as mean ± SEM (sorbitol vs sorbitol: n = 29, sorbitol vs agar: n = 22). **p*＜0.05, ***p*＜0.01, ****p*＜0.001, n.s: not significant; vs. day1; Steel-Dwass test (a, c, e), vs. “sorbitol vs sorbitol”; Steel-Dwass test (b).

**Sup. Fig. 6 Even with sucralose on both sides, the staying time in the proximity of the sorbitol food increased with time.**

(a-d) Behavioral changes in flies with time when fed sucralose + sorbitol food during daytime. (a) The change in staying time in the tube during the subjective daytime of day 1 and day 3. (b) The change in PDI. (c) Staying time at position 1 during the rest period and (d) the active period. Sorbitol was prepared at 5% concentration and dissolved in 1% agar + 1% sucralose. Each data is presented as mean ± SEM (sucralose + sorbitol vs sucralose + sorbitol: n = 63, sucralose + sorbitol vs sucralose: n = 52). ***p*＜0.01, ****p*＜0.001, n.s: not significant; vs. day1; Steel-Dwass test (a, c, e), vs. “sucralose + sorbitol vs sucralose + sorbitol”; Steel-Dwass test (b).

**Sup. Fig. 7 Starvation also increased the staying time in the proximity of sucrose.**

(a-e) Behavioral changes were measured after 48 hours of starvation when flies were fed with sucrose food during the subjective daytime of day 1. As a control group, we used flies fed with 5% sucrose + 1% agar for 48 hours. Staying time at position 1 (a), PDI to sucrose food (b), staying time at position 1 during the rest period (c) and the active period (d), and amount of sleep (e) sucrose was prepared at 5% and dissolved in 1% agar. Each data is presented as mean ± SEM (n = 32). n.s: not significant, ****p*＜0.001, Mann-Whitney U test (a-c, e), Student t test (d).

**Sup. Fig. 8 Consumption of sucrose also increased with prolonged starvation time.**

　Water food or sucrose food consumption after 24 and 48 hours of starvation (n = 20, 17, 21, 30). Each data is presented as mean ± SEM. ***p*＜0.01, ****p*＜0.001; Steel-Dwass test.
