# Supplementary material for: Preference of position in the proximity of various sugars revealed by location analysis of Drosophila melanogaster
Source: Sci Rep. 2024 May 17;14:11285. doi: 10.1038/s41598-024-61457-w (PMC11101431; doi:10.1038/s41598-024-61457-w)

Supplementary fig. 1

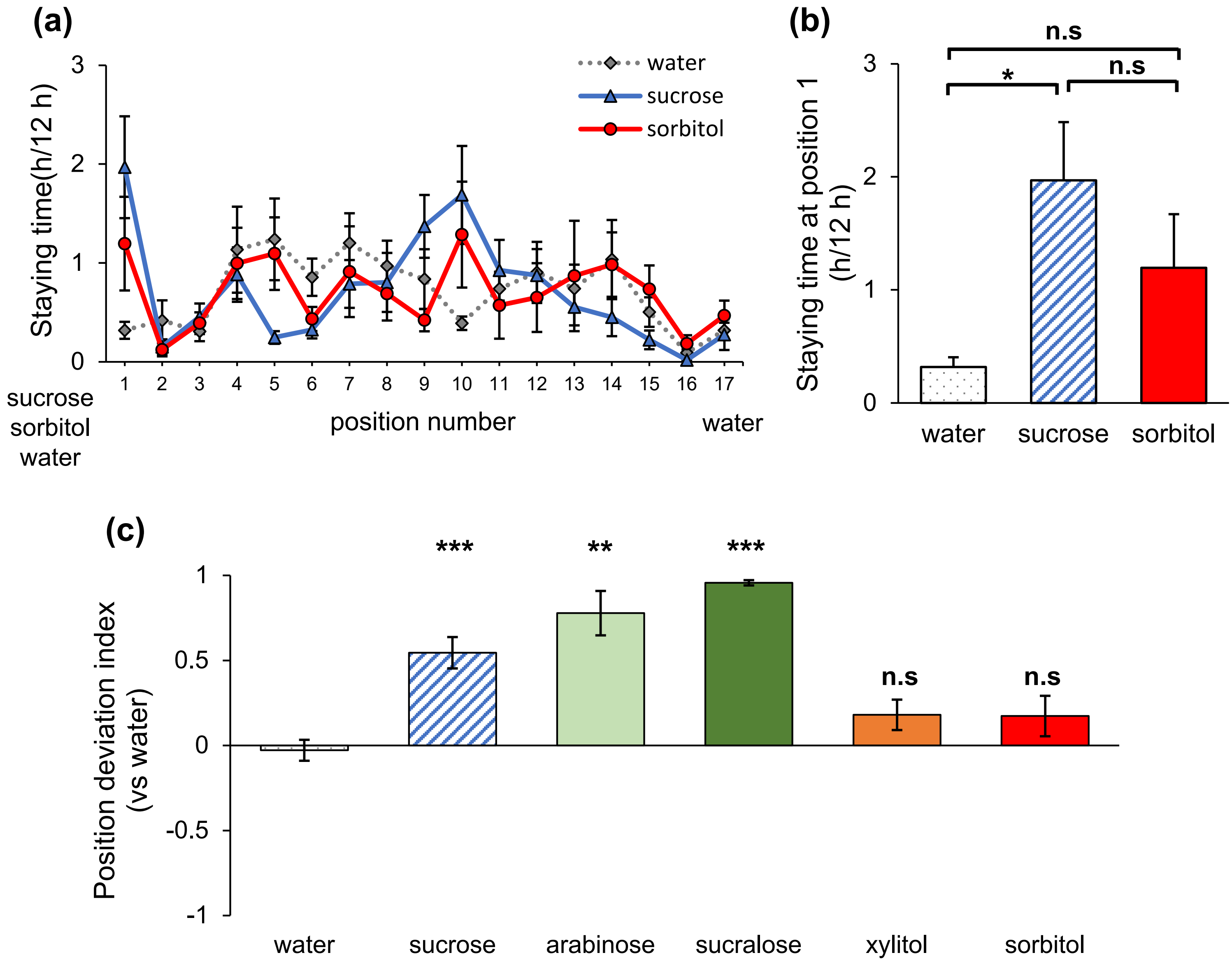

**Sweetness**	-	+	+	+	-	-
**Nutrition**	-	+	-	-	+	+

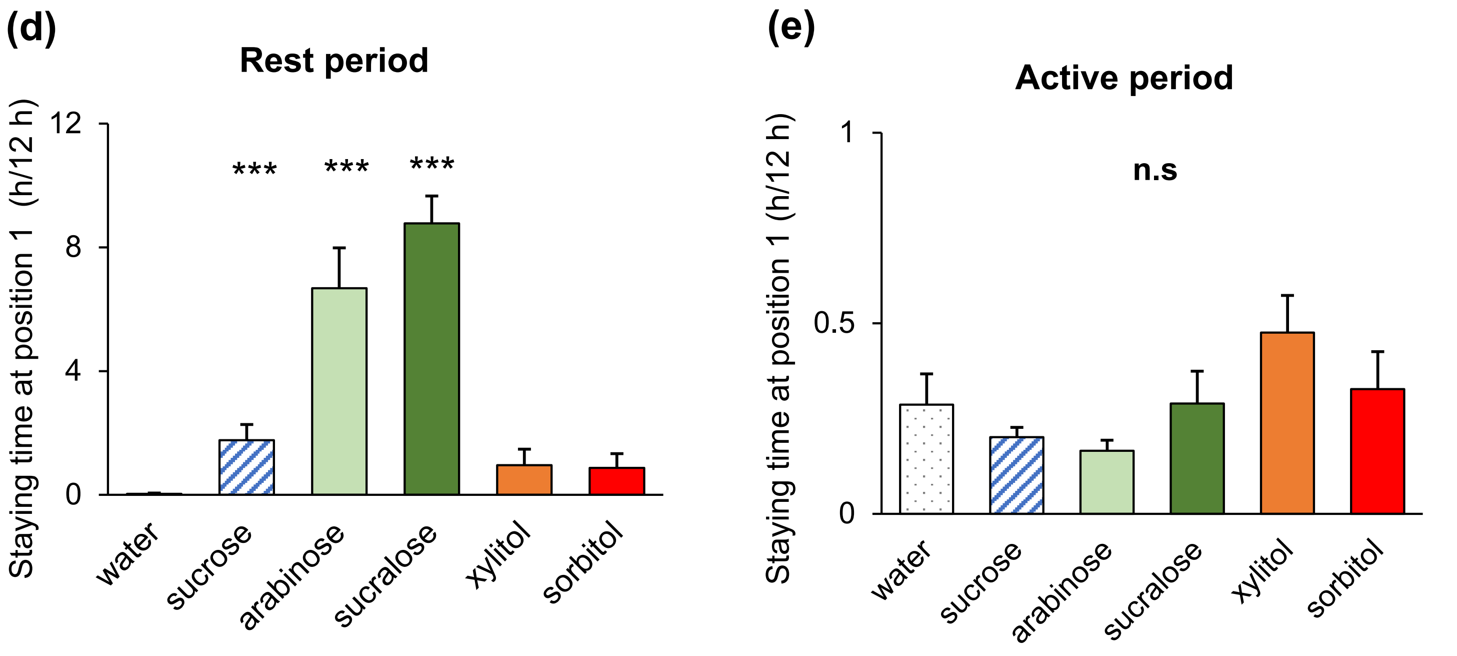

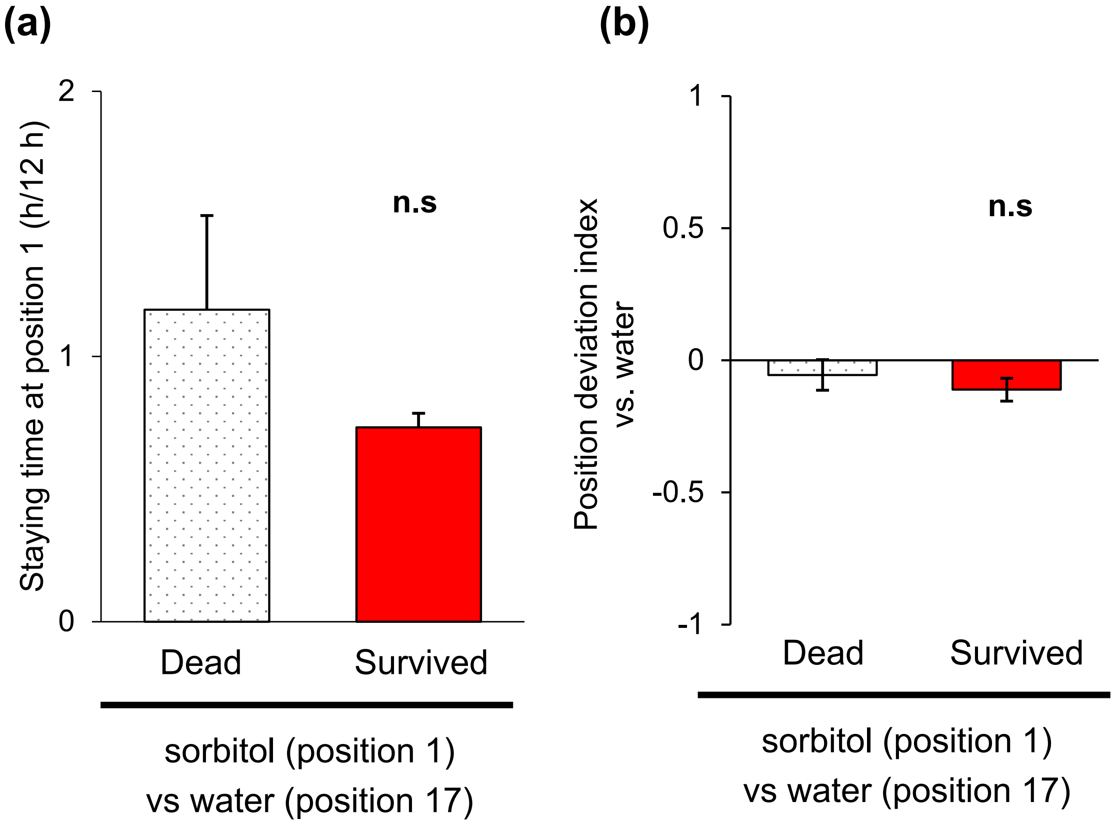
(a)
Staying time at position 1 (h/12 h)
2
1
0
n.s
Dead
Survived
sorbitol (position 1)
vs water (position 17)
(b)
Position deviation index
vs. water
1
0.5
0
-0.5
-1
n.s
Dead
Survived
sorbitol (position 1)
vs water (position 17)

Supplementary fig. 3

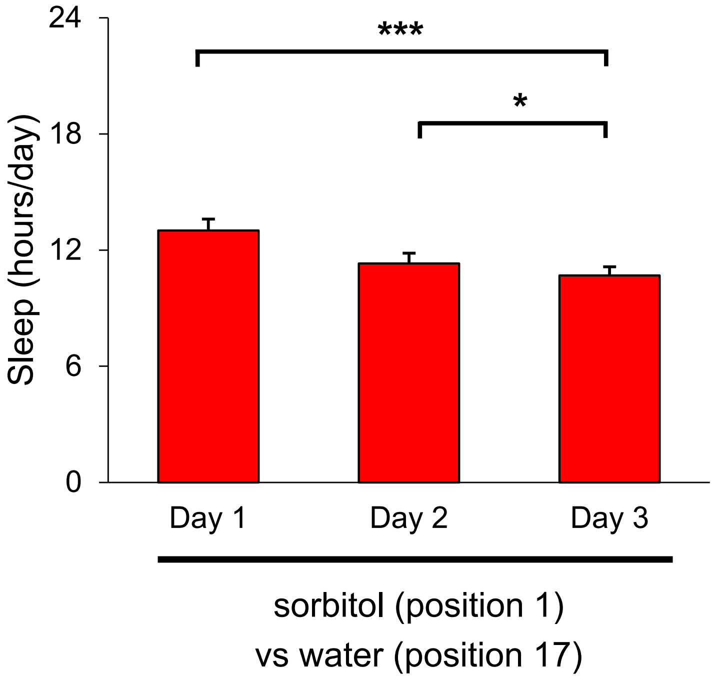

Supplementary fig. 4

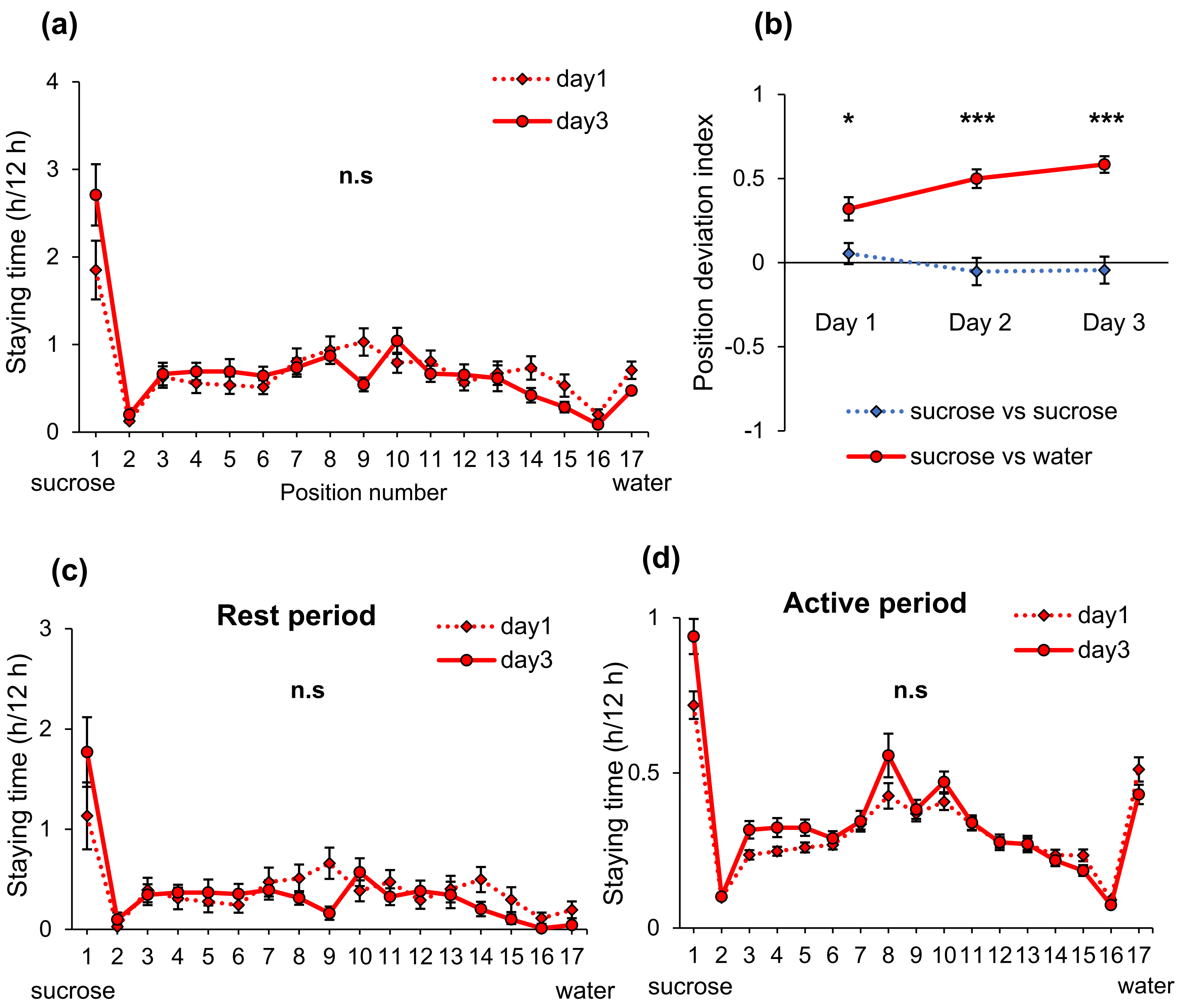

Supplementary fig. 5

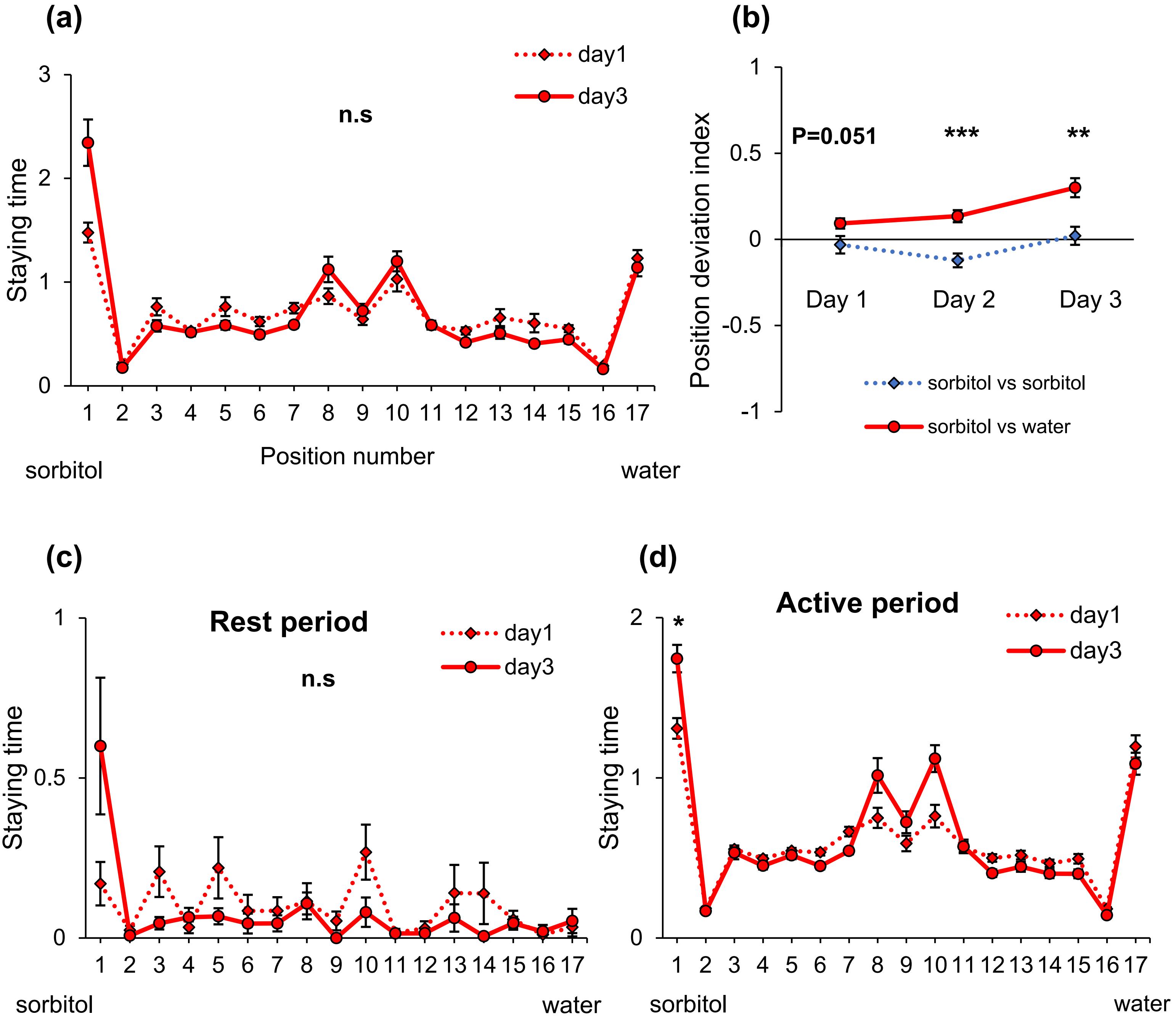

Supplementary fig. 6

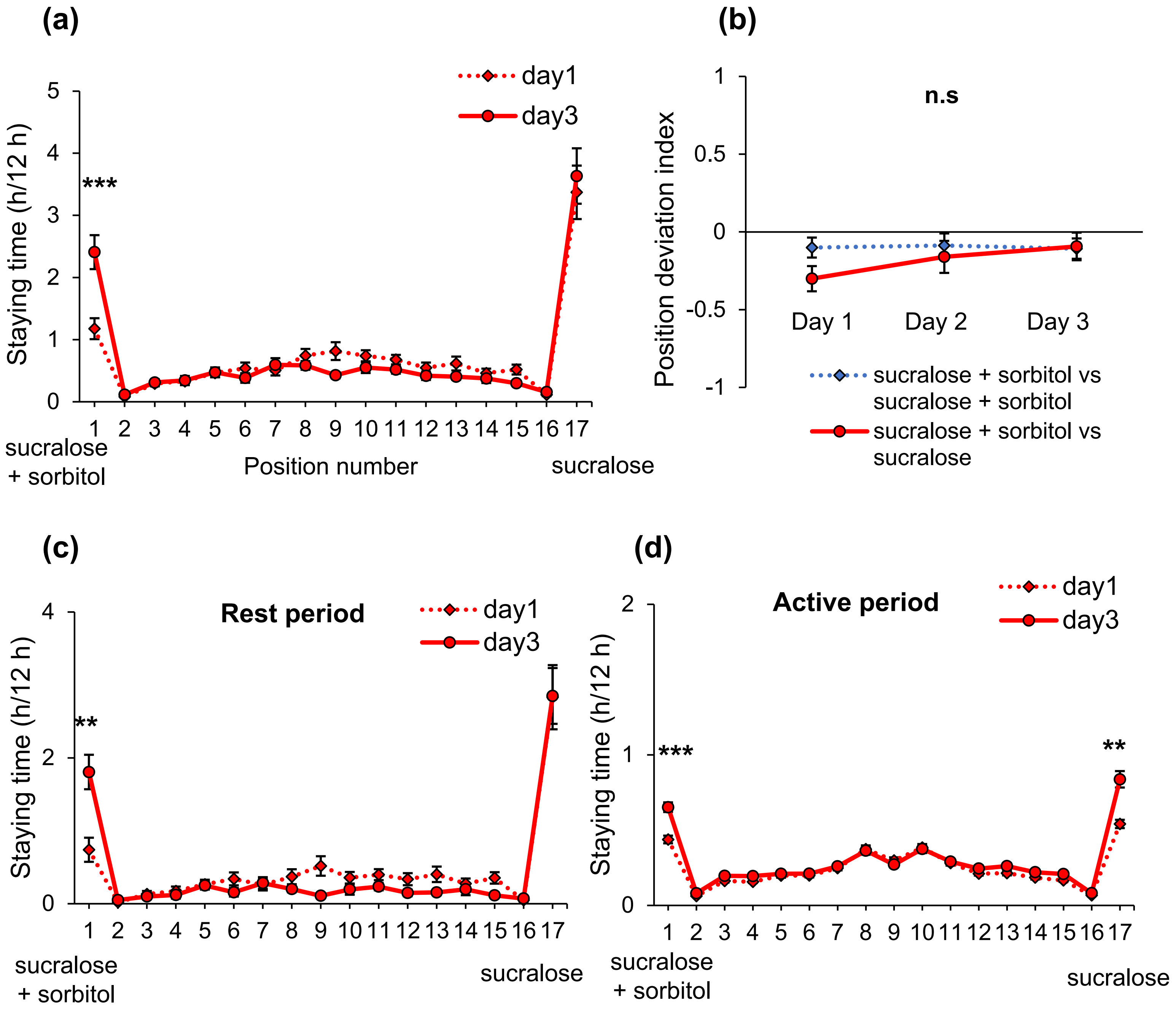

Supplementary fig. 7

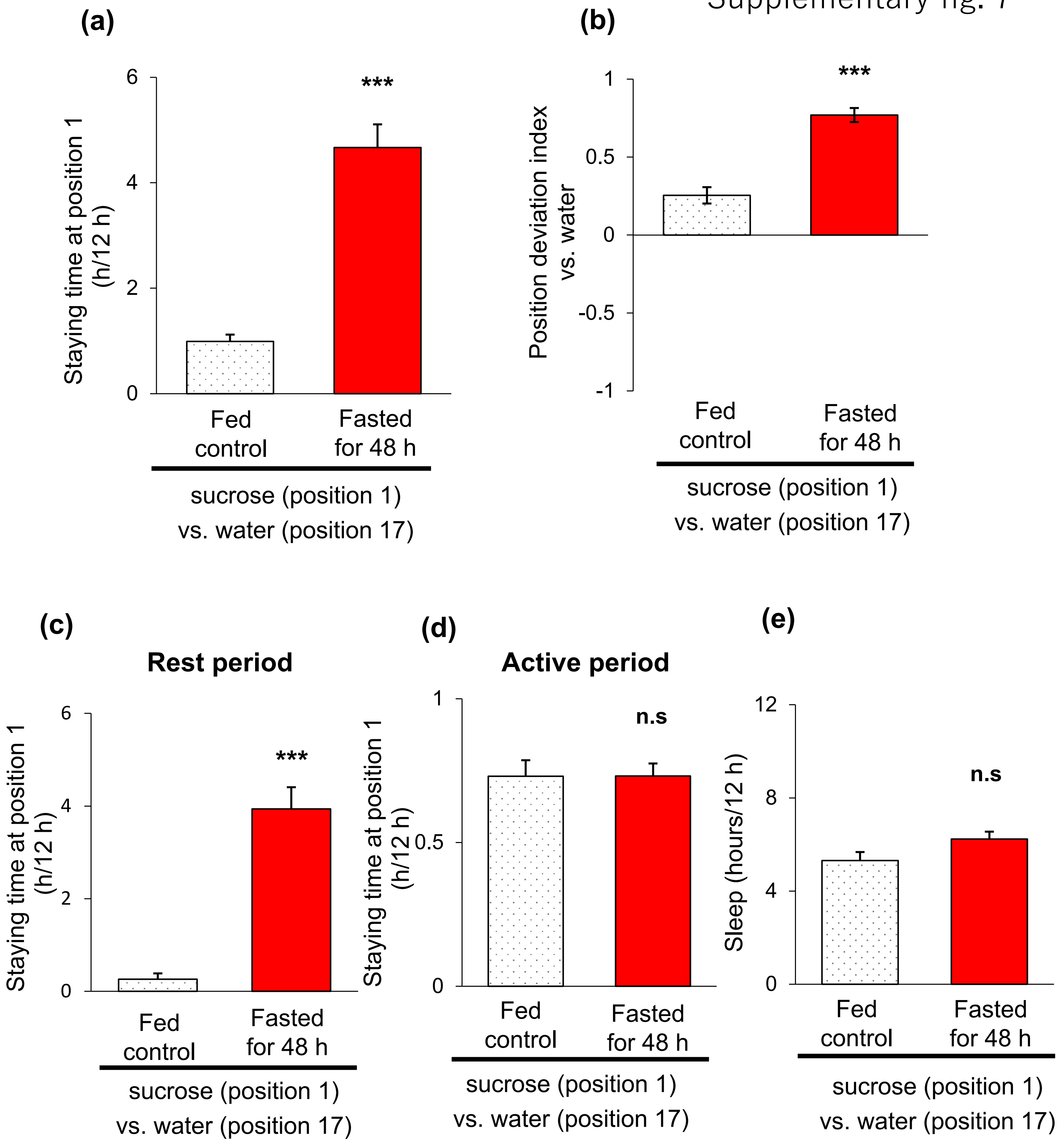

Supplementary fig. 8

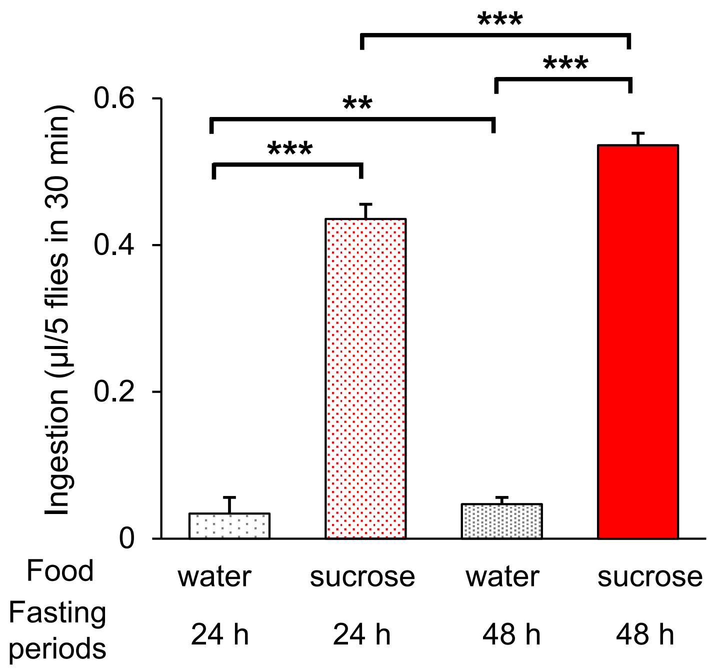

Supplement: Supplementary file 4 — Supplementary Figures. [file 41598_2024_61457_MOESM4_ESM.pdf]
